# Effect of Emissary Vein on Hemodynamics of the Transverse- Sigmoid Sinus Junction

**DOI:** 10.3389/fnhum.2021.707014

**Published:** 2021-11-12

**Authors:** Xiaoyu Qiu, Pengfei Zhao, Xiaoshuai Li, Heyu Ding, Han Lv, Zhenxia Mu, Xiaofei Xue, Shusheng Gong, Zhenghan Yang, Bin Gao, Zhenchang Wang

**Affiliations:** ^1^Department of Radiology, Beijing Friendship Hospital, Capital Medical University, Beijing, China; ^2^Faculty of Environment and Life, Beijing University of Technology, Beijing, China; ^3^Department of Otolaryngology Head and Neck Surgery, Beijing Friendship Hospital, Capital Medical University, Beijing, China

**Keywords:** pulsatile tinnitus, emissary veins, medical imaging, computational fluid dynamics, hemodynamics

## Abstract

**Objective:** To investigate the effect of the blood flow direction and afflux location of emissary veins (EVs) on the hemodynamics of the transverse-sigmoid sinus (TS-SS) junction.

**Methods:** A patient-specific geometric model was constructed using computed tomography venography (CTV) and 4D flow MR data from a venous pulsatile tinnitus (PT) patient. New EV models were assembled with the afflux at the superior, middle and inferior portions of the SS from the original model, and inlet and outlet directions were applied. Computational fluid dynamics (CFD) simulation was performed to analyze the wall pressure and flow pattern of the TS-SS junction in each condition.

**Results:** Compared to the model without EVs, the wall pressure was greatly increased in models with inlet flow and greatly decreased in models with outlet flow. The more closely the EV approached the TS-SS, the larger the pressure in models with inlet flow, and the smaller the pressure in models with outlet flow. The flow streamline in the lateral part of the TS-SS junction was smooth in all models. The streamlines in the medial part were regular spirals in outlet models and chaotic in inlet models. The streamlines showed no obvious changes regardless of afflux location. The velocity at the TS-SS junction of inlet models were uniform, medium-low flow rate, while in control and outlet models were the lateral high flow rate and the central low flow rate.

**Conclusion:** The flow direction and afflux location of EVs affect the hemodynamics of the TS-SS junction, which may influence the severity of PT.

## Introduction

Pulsatile tinnitus (PT) is a bothersome and not infrequent condition with vascular origins; this condition, mostly occurs in childbearing women. After a rigorous examination, venous origins are most commonly found (Lyu et al., 2018). In recent years, the newly treatable causes, innovative therapeutic procedures and possible mechanisms of venous PT have attracted a great deal of attention. The transverse-sigmoid sinus (TS-SS) junction has been reported as the most commonly involved site in venous PT. Recent studies have implied that venous PT may be generated by a combination of multiple vascular, skeletal and pressure factors (Eisenman et al., 2018). The roles of TS stenosis (Hewes et al., 2020), SS diverticulum (Lansley et al., 2017), venous outflow dominance, SS wall dehiscence (Zhao et al., 2016) and increased intracranial pressure (Funnell et al., 2018; Onder, 2018) have been well investigated. Increased wall pressure and a turbulent flow pattern at the TS-SS junction are thought to be associated with venous PT (Amans et al., 2018; Li et al., 2018; Mu et al., 2020), which implies that any procedure changing the hemodynamics in this area may alleviate or worsen the sound.

Emissary veins (EVs) are valveless channels between the dural venous sinuses and extracranial venous structures, although most of EVs are inflow, bidirectional flow and turbulence are possible under an increase in intracranial pressure (Mortazavi et al., 2012; Reardon and Raghavan, 2016). Most reports concerning EVs relate to the possibility of intraoperative iatrogenic bleeding (Ozen and Sahin, 2020). Three EVs communicate with the TS-SS junction or SS: the mastoid EV (MEV), the petrosquamosal sinus and the posterior condylar EV. These veins are found in more than 80% (Pekcevik et al., 2014; Gulmez Cakmak et al., 2019; Ozen and Sahin, 2020), 11.1% (Pekala et al., 2018), and 76.5% (Pekcevik et al., 2014) of the population, respectively, and their diameter varies from less than 1 mm to more than 5 mm. All three have been reported as causes of PT (Lambert and Cantrell, 1986; Lee et al., 2013; Liu et al., 2013), and the sound may be totally eliminated after ligation. However, the curative effect of EV ligation against PT is not stable in clinical practice. To our knowledge, studies on this topic are rare, and all of them are case reports. The roles of EVs in the occurrence of PT have not been investigated. It is important to explore which types of EVs may benefit from ligation for PT.

We speculate that EVs may result in the occurrence of PT. On the one hand, an abnormally enlarged EV may itself cause PT when presenting with both strong blood flow and a dehiscent wall along the mastoid air cells; on the other hand, it is more often for EVs to act as one of the most important factors to result in PT by changing the hemodynamics at the TS-SS junction (Kao et al., 2017). The effect of EVs may mainly depend on the direction and velocity of their blood flow and the location of the afflux. Thus, it is important to identify the roles of these factors in the changes on hemodynamics at the TS-SS junction to predict the effect of ligation.

Computational fluid dynamics (CFD) is an emerging technique to simulate hemodynamics in venous PT patients (Kao et al., 2017; Amans et al., 2018; Tian et al., 2019; Mu et al., 2020). This method utilizes computational processing to perform numerical analysis of fluid flow within three- dimensional (3D) models. These models can replicate patient-specific vascular segments by importing real geometrical information obtained by computed tomography venography (CTV) or magnetic resonance venography (MRV) into finite element analysis software (Markl et al., 2012).

CTV has been usually used to capture real vascular morphology (Narvid et al., 2011; Zhao et al., 2016), and 4D flow MR has the ability to capture accurate blood flow velocity and complex blood flow patterns *in vivo* (Kweon et al., 2016; Amans et al., 2018; Li et al., 2018). Through the combination of imaging examination and CFD analysis of individual finite element models, the simulation flow field can be visualized pictorially, and complex hemodynamic characteristics can be determined. However, CTV combined MR 4D-flow have not been adapted in CFD analysis.

In this study, based on CTV images and 4D flow MR velocity data of a patient with PT, 7 semipersonalized models were established, including an original model without EVs, 3 inlet EVs in the superior, middle and inferior portions of the SS, and 3 outlet EVs in the superior, middle and inferior portions of the SS. Steady-state CFD was used to investigate the hemodynamic effects of EV-related factors at the TS-SS junction. The wall pressure distribution, velocity streamlines and velocity difference at the TS-SS junction were calculated to assess the hemodynamic changes in the blood flow direction and afflux location of EVs at that junction. In this study, semi personalized simulation digital models were constructed based on multimodal image data to explore the influence of the EVs location and blood flow direction on the hemodynamics of TS-SS junction, so as to predict the possible role of EVs in PT.

## Materials and Methods

### Participant

Research ethics board approval was obtained in our institution. This study was based on a 22-year-old female who presented with a 2-year history of left-sided PT. The symptom was eliminated by compressing the ipsilateral jugular vein. The results of physical examination and otoscopic and audiometric evaluation were unremarkable. CTV showed an enlarged left MEV about 2 mm in diameter, ipsilateral upstream TS stenosis and SS wall dehiscence. PT was not relieved after ligation of the MEV.

### Imaging Features

Patient-specific raw CTV images were obtained using a 256-slice spiral CT scanner (Philips Medical Systems, Netherlands); the CTV data consisted of 231 slices acquired before ligation (512 × 512 pixels, 0.625 mm slice thickness) in Digital Imaging and Communications in Medicine format. Iodinated non-ionic contrast material was applied to display the lumens of the TS, SS, jugular vein, and large MEV on CTV images.

The patient underwent MRV and a 4D flow scan with a 3.0 T MR scanner (Philips, Ingenia, Netherlands). All visualization, assessment and interpretation of 4D flow data were performed using dedicated GT Flow 2.2.15 software (GyroTools, Switzerland).

### Vascular Model Construction

CTV image files were imported into MIMICS 20.0 software (Materialise, Belgium) to construct a 3D model of the left TS to jugular bulb containing the EV as original model, which was used to compare with the 3D model post-processed by 4D flow to verify the accuracy of CFD simulation; then a “ligation” model without EV was constructed as the control subject. Based on control subject, three new 20-cm-long and 2-mm-diameter EV models were built using Solidworks 2016 software (Dassault Systemes, United States). Due to the irregular shape of the patient’s real EV, there might be additional confounding factors; therefore, semipersonalized EV models with regular shapes were simulated to control unrelated variables. The new finite element models were assembled at the superior, middle and inferior portions of SS, and the direction were set to inflow (group 1) and outflow (group 2). The presence of superior, middle, and inferior inlet EVs and the presence of superior, middle, inferior outlet EVs were named cases 1–6, respectively. Through image segmentation and 3D model creation, geometric models were obtained. The models were smoothed using Freeform and Geomagic software (Geomagic, United States). After processing, the surface geometries were saved in standard tessellation language format.

### Computational Models and Simulations

**FIGURE 1:**
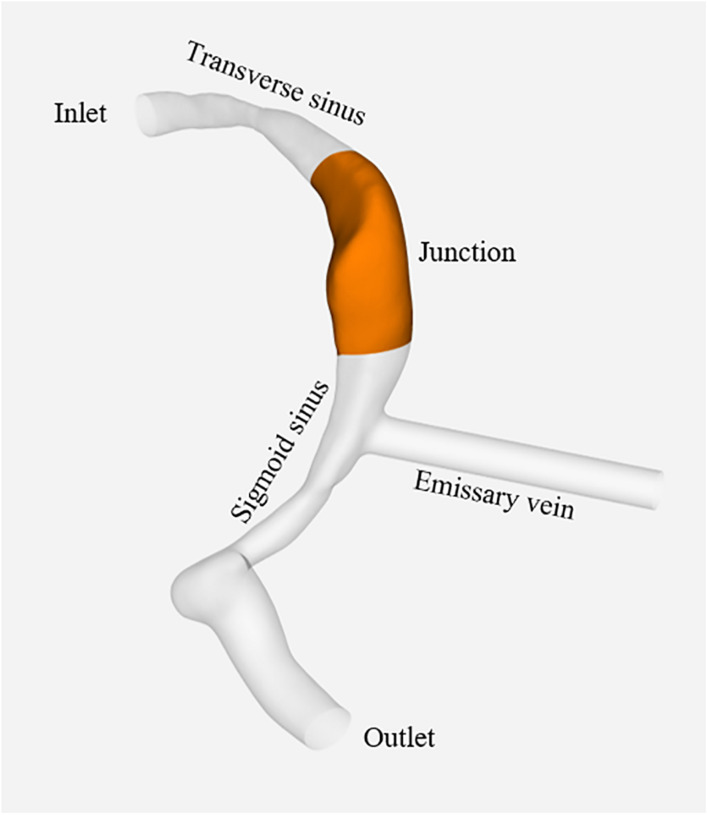
Positions of inlet, TS, TS-SS junction, SS, EV, and outlet.

The standard tessellation language format files were imported into Fluent 2019 R1 (ANSYS, Inc., Cecil Township, Pennsylvania, United States) for meshing; high quality polyhedral 3D meshing was successfully created. In order to confirm the adaptive grid size, the wall pressure was used as the criterion for the grid-independence test. The area of wall pressure was located at the TS-SS junction (Figure 1). A grid with less than 5% pressure error was considered acceptable. A maximum of 1013699 elements were developed, which was a sufficient number for this study. The element size of each case was set to a maximum of 0.2 mm. Four boundary layers were generated to resolve the flow field at the fluid-wall interface. The venous wall was assumed to be rigid and a no-slip boundary condition was applied. The blood was assumed to be a laminar and incompressible Newtonian fluid with a density of 1050 kg/m^3^ and a viscosity of 0.0035 Pa/s. The constant inlet section was located at the TS of the model, and the boundary condition used the real blood flow velocity of 45 cm/s at the TS measured by 4D flow; the constant outlet was located at the jugular bulb, and the boundary condition was set to an absolute pressure of 0 pa. In the original model, since the real blood flow in the EV was observed as the outflow direction in 4D flow, the boundary condition was set to the real velocity of −20 cm/s measured by 4D flow. According to the flow directions prescribed by the study design, when the distal newly built EVs were used as inlets or outlets, the boundary conditions were ± 20 cm/s, respectively. The Navier-Stokes formula, solved with Fluent 2019 R1, was used as the governing equation for calculations. Based on the simulation results, several flow parameters were calculated and color-coded according to magnitude and distribution to evaluate the effects of model hemodynamics quantitatively. These parameters included wall pressure distribution, wall maximum pressure (P_max_), wall average pressure (P_avg_), velocity streamlines and distribution, maximum velocity (V_max_) and average velocity (V_avg_). The original model simulation only needed to obtain the velocity streamline, which was used to compare with the 4D flow results.

## Results

### Comparison of Original Model Computational Fluid Dynamics Simulation and 4D Flow Results

**FIGURE 2:**
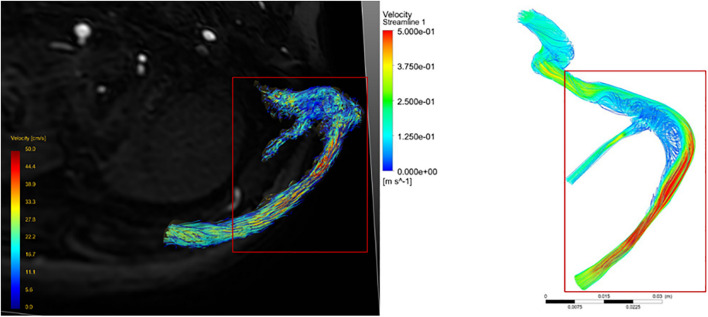
Comparison of velocity streamline obtained from 4D flow MR post-processing with that simulated by CFD.

Comparing the velocity streamline of the real 4D flow MR postprocessing model of the patient collected in this study with the streamline model obtained by CFD simulation, it was found that the blood flow pattern and velocity were consistent (Figure 2), which confirmed the accuracy of CFD simulation *in vivo* blood flow state. Therefore, the follow-up results of this study can be considered as reliable.

### The Control Subject

**FIGURE 3:**
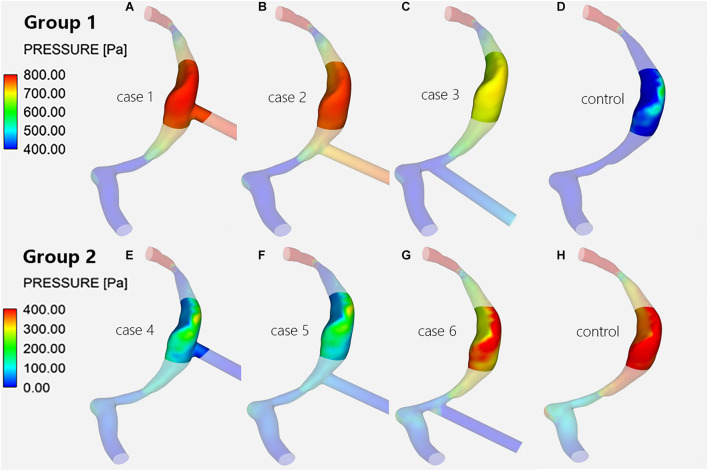
Wall pressure distribution of the TS-SS junction. The upper row shows the wall pressure distribution of models with inlet EVs **(A–C)** and without EVs **(D)** in group 1; the lower row shows that of models with outlet EVs **(E–G)** and without EVs **(H)** in group 2.

**FIGURE 4:**
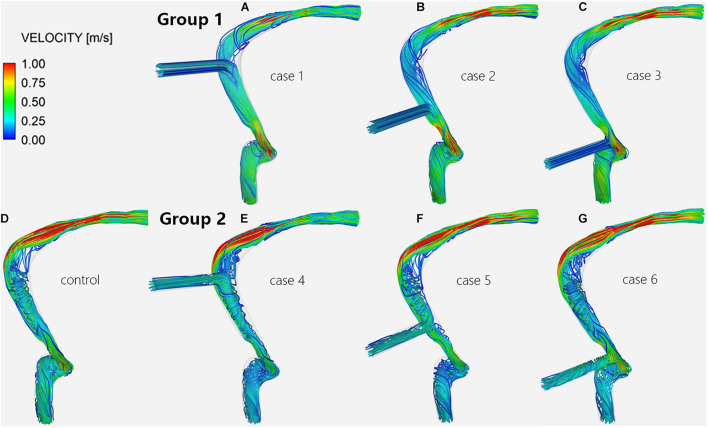
Velocity streamlines of the TS-SS junction. The upper row shows the velocity streamlines of inlet EVs **(A–C)** in group 1; the lower row shows those of models without EVs **(D)** and with outlet EVs **(E–G)** in group 2.

**FIGURE 5:**
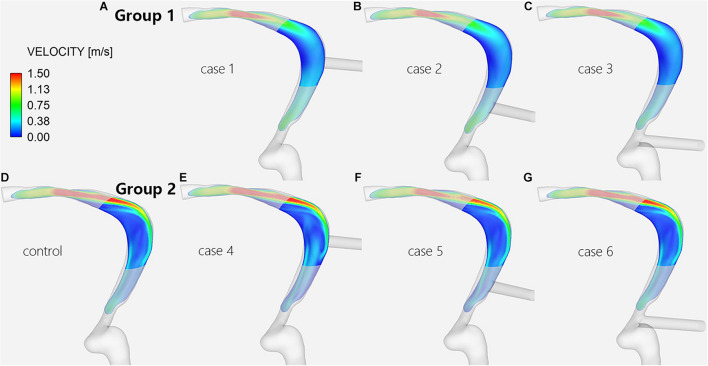
Velocity distribution of the TS-SS junction. The upper row shows the velocity distribution of inlet EVs **(A–C)** in group 1; the lower row shows those of models without EVs **(D)** and with outlet EVs **(E–G)** in group 2.

The CFD simulation results of the control subject, which can be compared with the results of cases 1–6, are shown in Figures 3D,H. The pressure at the TS-SS junction area was lower than the pressure upstream of the transverse sinus stenosis and was higher at the lateral wall of the TS-SS junction, especially at the dome (Figures 3D,H). The shape of the streamlines was smoother on the lateral side, compared to regular spiral on the medial side. The overall flow pattern at the junction area was regular (Figure 4D). The velocity distribution illustrated that the velocity at the junction area was significantly slower than the velocity in the TS stenosis. The flow velocity on the lateral side of the junction area was faster than the velocity on the medial side (Figure 5D).

### Wall Pressure Changes at the Transverse-Sigmoid Sinus Junction

When there was influent blood flow in the EVs, whether they were located at the superior, middle, or inferior portion of the SS, the wall pressures of group 1 at the junction were higher than those of the control subject, with the difference being most notable at the lateral wall (Figures 3A–D); when there was reverse flow, the pressures of group 2 were lower than those of the control subject, but the pressures was still higher at the dome of the lateral wall than at the medial wall at the TS-SS junction (Figures 3E–H).

**FIGURE 6:**
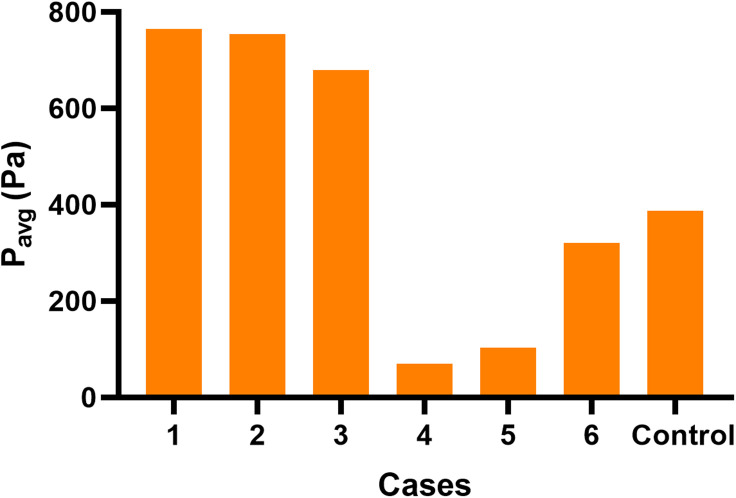
Wall P_avg_ at the junction area in each case.

In group 1, the pressures at the TS-SS junction decreased gradually as the EVs moved down, but they were higher than those of the control subject (Figures 3A–D). Conversely, in group 2, as the EV position moved down, the pressures at the TS-SS junction increased, but they were lower than those of the control subject (Figures 3E–H). Again, the highest pressures were always at the dome of the TS-SS junction. Figure 6 illustrates the P_avg_ on the junction area, and the quantitative P_max_ and P_avg_ are shown in Table 1.

**TABLE 1:** Quantitative data on wall pressures and velocities.

**Case**	**Model**	**P_max_(Pa)**	**P_avg_(Pa)**	**V_max_(cm/s)**	**V_avg_(cm/s)**
Control	No EV	593.6	386.7	148.6	25.7
1	s-in	791.2	764.8	63.3	18.2
2	m-in	780.1	754.3	69.0	16.9
3	i-in	706.9	679.3	70.6	16.7
4	s-out	307.1	70.1	150.0	26.6
5	m-out	304.3	103.1	147.1	25.3
6	i-out	521.0	320.1	149.6	25.3

*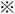 s, superior; m, middle; i, inferior; in, inlet; out, outlet; P_max_, wall maximum pressure; P_avg_, wall average pressure; V_max_, maximum velocity; V_avg_, average velocity.*

### Blood Flow Patterns at the Transverse-Sigmoid Sinus Junction

Regardless of whether the EVs were inlets or outlets, the blood flow patterns of cases 1–6 compared with the control subject showed that the streamlines in the lateral part of the TS-SS junction area were all smooth (Figure 4). The streamlines of group 1 in the medial part were slightly disordered (Figures 4A–C), while those of group 2 were regular spirals similar to the control subject (Figures 4E–G).

**FIGURE 7:**
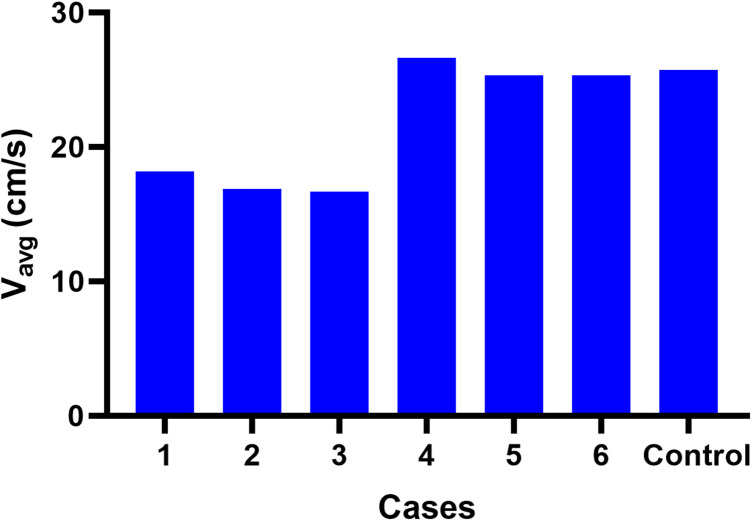
Blood flow V_avg_ at the junction area in each case.

In group 1, the changes in blood flow velocity distribution at the TS-SS junction showed little change, but they were different from those of the control subject, and the flow velocities were relatively uniform, medium-low flow rate (Figures 5A–D). In group 2, the flow velocity changes at the TS-SS junction were similar to those of the control subject, group 2 had higher flow velocities in the lateral and medial parts than lower velocities in the central part, similar to the control subject (Figures 5D–G). The overall flow velocity of TS-SS junction in group 1 was lower than that in group 2. Figure 7 indicates the difference in V_avg_ in each case. The quantitative data on V_max_ and V_avg_ are also shown in Table 1.

## Discussion

This study is the first to comprehensively explore the effect of EV on the hemodynamics of the TS-SS junction. Finite element analysis, a powerful simulation tool in biomechanics (Miller, 2009), was used to show the influence of the flow direction and afflux location of EVs on the hemodynamics of the TS-SS junction. Previous studies of the hemodynamics of venous PT mostly used CTV or digital subtraction angiography to construct CFD models (Kao et al., 2017; Amans et al., 2018; Tian et al., 2019; Mu et al., 2020). To evaluate the effects of EVs in different locations and different flow directions on the hemodynamics of the TS-SS junction, we included CTV combined with 4D flow images from a venous PT patient to construct a highly simulated CFD model. In previous studies, data on the inlet blood flow of the TS, which we consider important for the results, were mostly assigned subjectively. In this study, 4D flow MR was used to obtain the true velocity at the entrance and to compare the flow pattern with the initial model to improve the reliability of the results.

As our subject, we chose a patient with the most common features of venous PT, such as ipsilateral TS stenosis (Han et al., 2017; Lansley et al., 2017; Eisenman et al., 2018; Hewes et al., 2020), distention of the TS-SS junction (Hsieh and Wang, 2020), SS wall dehiscence (Dong et al., 2015; Mundada et al., 2015; Eisenman et al., 2018), and ipsilateral EV (Dong et al., 2015), so that the study would reflect common clinical situations. The diameter of the EVs was set to 2 mm instead of a much larger size for the same reason. The location was set to three different positions to simulate the afflux locations of the petrosquamous sinus, MEV and posterior condylar EV. Bidirectional blood flow was permitted because the EVs are valveless. In addition, we assumed that the faster the velocity, the more remarkable the abovementioned effects would be; therefore, this important factor was not specifically investigated here.

Pressure change has been reported as an important indicator affecting PT (Mu et al., 2020). Our results showed that the flow directions of EVs may significantly affect the pressure of the TS-SS junction. We concluded that the EV flow direction must be considered when choosing the ligation operation. If there is strong blood flow from an EV into the SS, ligation may be helpful to alleviate the sound. Otherwise, this treatment will be not only worthless but also harmful. For example, ligation may increase the risk of intracranial hypertension and associated serious neurological injury if the blood flow blocked by the ligation cannot be compensated (Zhao et al., 2016). When an EV acts as an inlet, the vessel wall at the TS-SS junction is subjected to the impact of blood flow from at least two directions, including one from the TS and another from the EV, which may increase the pressure. Under these circumstances, EV ligation is equivalent to reducing the blood flow into the SS, and the pressure is reliably reduced as well. In contrast, When an EV is the outlet, as a collateral drainage vein pathway, it can prevent any increase in upstream resistance, thus affecting the overall upstream pressure gradient and limiting the increase in blood pressure (Levitt et al., 2016). Therefore, after ligation, the area of the downstream outlet decreases, which will definitely increase the pressure at the SS. In addition, when the EV is inlet, the higher the EV position is, the closer it is to the TS-SS junction, and the greater the blood flow impact from both sides of TS and EV, so the higher the pressure is. The ligation effect may be better at this time.

TS venous flow sharply turned at the TS-SS junction, affecting the lumen and inducing an evident vortex at the TS-SS junction (Shin et al., 2019). Our findings suggested that the blood flow patterns at the junction in all models had smooth streamlines on the lateral side. The angle between the TS and SS influences the distribution of the flow field; therefore, the difference in the flow field may be caused by the different shapes of individual vessels. In this case, because of the angle between the TS and SS, the blood flow from the TS is concentrated on the lateral side, while the flow velocity on the medial side is relatively low. Our results also suggested that the flow velocity in the inlet group was much lower than in the outlet group and the control subject. The reason may be associated with that the EV inlet blood flow opposes the TS inlet blood flow, and the kinetic energy is transformed into potential energy according to mass conservation, resulting in increased wall pressure and decreased flow velocity. And in the inlet group, EV blood flowed into the SS, the normal lateral high-speed blood flow band disappeared after impact, replaced by a relatively uniform, slightly increased flow velocity distribution in central of the TS-SS junction. The medial part of EV models with outlet EVs or without EVs is a regular spiral flow, which may be related to the swirling flow of blood under normal circumstances (Ha et al., 2015); the irregular flow on the medial side of inlet EV models may be caused by the collision of opposite blood flow, which would cause the normal swirling flow to disappear and be replaced with slightly disordered blood flow. It is also concluded that ligation should be considered when the large EV of a PT patient is an inlet.

Unfortunately, in this original case, PT still exists after large EV ligation. Part of the reason is that the EV is located in the middle to inferior portion of the SS, which is a non-negligible distance from the TS-SS junction, and 4D flow MR postprocessing images showed that there was outlet blood flow in the EV. According to the abovementioned research, this kind of EV has little effect on the hemodynamics of the TS-SS junction. Therefore, ligation is not an effective treatment for the patient, which is consistent with the results of the present study.

This study has several limitations. First, there is only one case, which is not completely representative, although the most common morphologic and hemodynamic characteristics of EV and TS-SS junction in PT patients were set as numerical boundary conditions. Second, this case is a semi-individual model. The disadvantage of the semipersonalized model is that it cannot fully simulate the characteristics of the original structure and can implement only targeted simulations. The research model requires different EV positions; thus, in order to control other influencing factors, EV models with the same regular shape are established, and the other parts are personalized. Third, because the venous sinus is limited to the skull and dura mater, and is less elastic than an artery, we considered it acceptable to neglect wall elasticity, and the vessel wall of the venous sinus was assumed to be rigid and governed by the no-slip boundary condition (Sforza et al., 2010). Fourth, the results show the influence of the MEV entry distance on the blood flow at the junction, but the relationship between the degree of the effect on blood flow and the distance was not quantified, and the specific orientation of the entry point was not considered. Finally, this study focused only on the effects of different EV positions and flow directions on the hemodynamic of the TS-SS junction. In fact, the EV diameter and internal flow rate can also affect the target area; this influence will be further studied in the future.

## Conclusion

A specific-patient hemodynamic analysis implies that the flow direction and afflux location of EVs affect the hemodynamics of the TS-SS junction and the severity of PT. The main observation is that when the enlarged EV is located in the superior SS and the blood flow through the EV is positive, the hemodynamic indicators of the TS-SS junction area are markedly worse than in the model without EV. Venous PT patients who have enlarged EVs adjacent to the TS-SS junction and inlet flow are most likely to benefit from ligation. The combined use of imaging technology and CFD simulation to obtain hemodynamic information, such as wall pressure and flow velocity, can provide a more accurate inference regarding the etiology of PT for clinical practice and establish a theoretical basis for subsequent treatment.

## Data Availability Statement

The original contributions presented in the study are included in the article/supplementary material, further inquiries can be directed to the corresponding author/s.

## Ethics Statement

The studies involving human participants were reviewed and approved by the Beijing Friendship Hospital, Capital Medical University. The patients/participants provided their written informed consent to participate in this study.

## Author Contributions

XQ, XL, HD, and HL collected the clinical and imaging data. XQ, ZM, XX, and PZ performed the experiment and drafted the manuscript. ZW and BG designed the study and ensured the questions related to all aspects of the work. PZ, ZY, BG, and ZW gave critical comments on the manuscript. All authors contributed to the article and approved the submitted version.

## Conflict of Interest

The authors declare that the research was conducted in the absence of any commercial or financial relationships that could be construed as a potential conflict of interest.

## Publisher’s Note

All claims expressed in this article are solely those of the authors and do not necessarily represent those of their affiliated organizations, or those of the publisher, the editors and the reviewers. Any product that may be evaluated in this article, or claim that may be made by its manufacturer, is not guaranteed or endorsed by the publisher.
